# Numerical Prediction of Residual Stresses Distribution in Thin-Walled Press-Braked Stainless Steel Sections

**DOI:** 10.3390/ma13235378

**Published:** 2020-11-26

**Authors:** Ayad Mutafi, Noorfaizal Yidris, Seyed Saeid Rahimian Koloor, Michal Petrů

**Affiliations:** 1 Department of Aerospace Engineering, Universiti Putra Malaysia, Selangor 43400, Malaysia; ayad2motafi@live.com; 2 Institute for Nanomaterials, Advanced Technologies and Innovation (CXI), Technical University of Liberec (TUL), Studentska 2, 461 17 Liberec, Czech Republic; s.s.r.koloor@gmail.com (S.S.R.K.); michal.petru@tul.cz (M.P.)

**Keywords:** stainless steel, finite element, 3D-FE, 2D-FE, residual stress, compressive stress, tensile stress

## Abstract

Stainless steels are increasingly used in construction today, especially in harsh environments, in which steel corrosion commonly occurs. Cold-formed stainless steel structures are currently increasing in popularity because of its efficiency in load-bearing capacity and its appealing architectural appearance. Cold-rolling and press-braking are the cold-working processes used in the forming of stainless steel sections. Press braking can produce large cross-sections from thin to thick-walled sections compared to cold-rolling. Cold-forming in press-braked sections significantly affect member behaviour and joints; therefore, they have attained great attention from many researchers to initiate investigations on those effects. This paper examines the behaviour of residual stress distribution of stainless steel press-braked sections by implementing three-dimensional finite element (3D-FE) technique. The study proposed a full finite element procedure to predict the residual stresses starting from coiling-uncoiling to press-braking. This work considered material anisotropy to examine its effect on the residual stress distribution. The technique adopted was compared with different finite element techniques in the literature. This study also provided a parametric study for three corner radius-to-thickness ratios looking at the through-thickness residual stress distribution of four stainless steels (i.e., ferritic, austenitic, duplex, lean duplex) in which have their own chemical composition. In conclusion, the comparison showed that the adopted technique provides a detailed prediction of residual stress distribution. The influence of geometrical aspects is more pronounced than the material properties. Neglecting the material anisotropy shows higher shifting in the neutral axis. The parametric study showed that all stainless steel types have the same stress through-thickness distribution. Moreover, R/t ratios’ effect is insignificant in all transverse residual stress distributions, but a slight change to R/t ratios can affect the longitudinal residual stress distribution.

## 1. Introduction

Stainless steel structural members have a wide range of civil applications, especially in highly corrosive environments. In addition to excellent durability, stainless steel has a number of beneficial physical features, for example, high strength, ductility, and stiffness, making it suitable for construction. Cold-rolling and press-braking are the most cold-working processes used in forming stainless steel sections. Press braking can produce larger cross-sections from thin to thick-walled sections compared to cold-rolling.

Cold-forming in press-braking sections may significantly influence the member performance. This fact has caught the attention of researchers to investigate those effects. Measurement of the residual stresses in the longitudinal direction of thin-walled cold-formed steel open sections using the sectioning method showed that higher residual stresses in the corner zones than in flat zones [1]. Weng and White [2] investigated the amount of residual stresses in transverse direction for press-braked thick-walled steel sections. They presented the residual stresses distribution in the plate through-thickness as a zigzag distribution. Weng and Pekoz [1] proposed a distribution model for the longitudinal residual stress. The entire internal and external surfaces of a cold-formed section had 0.5 f_y_ tension stresses and 0.5 f_y_ compression stresses, respectively, where f_y_ denoted as the nominal yield stress. Schafer and Pekoz [3] gathered and evaluated residual stress data in press-braked open sections and presented various residual stresses values for section’s bending and flat zones.

For cold-formed stainless steel sections, systematic experimental investigations have been performed for welded [4,5] and cold-rolled [6,7,8] sections. In contrast, very few experimental studies have been carried out on press-braked sections. A model was suggested by Cruise and Gardner [8] and Gardner and Cruise [9] for residual stress distribution in the longitudinal direction for press-braked stainless steel angle members. They measured the stresses by using the sectioning method. They assumed that the form of residual stress distribution was linear or rectangular stress block due to the difficulty at that time in measuring the longitudinal residual stress in through-thickness direction.

Laboratory measurement (destructive and non-destructive types) of residual stresses is labor-intensive, time-consuming, and incurs a lot of material wastage. Thus, many theoretical efforts have been performed to predict residual stresses. Rondal [10] predicted residual stresses at bending zone of cold formed sections using bending simulation of a steel sheet. He used the Prandtl-Reuss flow rule and the von Mises yield criterion to present his numerical algorithm. Quach et al. [11,12,13,14], conducted systematic theoretical research on residual stresses in press braked carbon steel channel sections as well as stainless steel, and suggested a sequence of models for predicting residual stress accurately. Relevant factors related to the allocation of residual stresses (e.g., the entire production cycle, neutral axis shift, material anisotropy, and the full range stress-strain curve) were considered. Moen et al. [15] suggested a mechanics-based forecast technique for determining significant plastic strain and residual stresses in cold-rolled steel components. They neglected certain variables that had insignificant impacts on the final residual stresses and deriving a straightforward close-formed alternative. Amouzegar et al. [16] established an incremental algorithm to investigate the distribution of residual stresses and residual strains through plate thickness of open sections (e.g., open channels) that have experienced the forming process. Their results proved that the corner radius is the most critical parameter, which affects residual stresses and residual strains at the corner regions. The yield stress and coil radius play significant roles in the residual stresses and strains variation of the flat regions. A simplified model of residual stress distribution proposed by Zheng et al. [17]. They established a simplified model based on analyzing the key mechanisms in the press-braking. The proposed model was assumed as a two-stage (bending and rebounding) plane strain and a pure bending process. The study concluded the validity of the proposed simplified model for press braked stainless steel sections with *r*_i_/*t* ratios greater than 2.0.

There are several techniques of numerical solutions for sheet metal forming; however, the finite element method has become the most favored technique to solve sheet metal forming problems due to its accuracy [18]. Finite element investigations have been adopted for predicting residual stresses of sections made from the roll-forming process as in [19,20]. Larrañaga [20] established a 3D-FE simulation to calculate the forces and torques during the roll-forming process. Abvabi et al. [19] studied the roll-forming of a V-section experimentally and numerically. They concluded that the quality of produced sections (e.g., cold-rolling) can be affected by the magnitude of residual stresses in the material.

**Figure 1:**
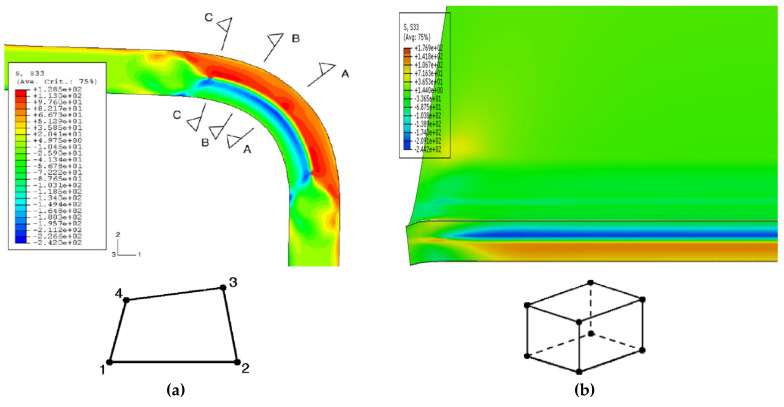
Longitudinal residual stress distribution prediction and element types adopted in the prediction: (**a**) 2D-FE technique [12], (**b**) 3D-FE technique [24].

Many numerical studies have examined the influence of the roll-forming process on steel member behaviour. This is because the roll-forming operation is appropriate for mass production. These studies have employed different finite element procedures (implicit, dynamic explicit) and elements (shell element and solid element) to achieve their objectives. In contrast, the press-braking operation considers cost-effective and economical for low volume productions. Despite that, a limited number of numerical studies has investigated residual stress prediction in press-braking such as in [12,13,14,17]. These investigations have confirmed the variation of residual stresses through thin-walled steel thickness using a similar 2D FE model. They applied a 2D plane strain element with 4-noded and assumed that each node has four degrees of freedom (two translations and two rotations). Figure 1a shows the longitudinal residual stress predicted using the finite element-based method introduced by Quach et al. [12]. They predicted residual stress at the flat region using an analytical solution for coiling-uncoiling process proposed by them. Then, they predicted residual stress from press-braking by implementing a 2D-FE technique. Later, Quach et al. [21] observed residual stress variation near the corner ends along the member length but had not clarified the reason for this non-linear stress distribution. Then, Abambres and Quach [22] stated that the prediction of residual stress along the corner region is neglected in the literature due to the lack of investigations experimentally and numerically in order to verify the stress distribution along the region. Gerbo et al. [23] performed 2D and 3D finite element modelling (FEM) to validate the new non-destructive approach measuring residual strains of cold-bent thick plate steel. They recommended a 3D-finite element for detailed investigation. Based on that, Mutafi et al. [24] investigated residual stresses of thin-walled press-braked cold-formed carbon steel in the longitudinal direction using the 3D-FE technique. They used a 3D solid element with 8-nodes where each node has six degrees of freedom (three translations and three rotations) as shown in Figure 1b. They stated in their conclusion that residual stresses near corners have different variations. In addition, Mutafi et al. [25] investigated the surface longitudinal residual strains of thin-walled press-braked cold-formed steel and showed that the longitudinal residual strain (compressive and tensile strain) near the edge is greater than the rest of the corner region.

Therefore, this paper aims to extend the investigation of the distribution of residual stresses along the bending region of stainless steel press-braked sections by implementing the same technique in [24,25] giving due consideration to the influence of coiling-uncoiling process. The study proposed a full finite element procedure to predict residual stress starting from coiling-uncoiling to press-braking. In addition, studies such as [4,26] stated that material anisotropy effect is neglected; therefore Zheng et al. [17] validated their simplified method for stainless steel by adopting a 2D-FE technique with an assumption that the stainless steel specimen is isotropic. Comparison with 2D-FE technique presented in Quach et al. [14] demonstrated some notable differences. Hence, this paper presents a comparison between 3D-FE and the 2D-FE methods presented in [14,17] to examine each model’s capabilities with regards to the determination of residual stresses.

This study provided a parametric study with regards to the through-thickness residual stress distribution of stainless steels (i.e., ferritic, austenitic, duplex, lean duplex). Each stainless steel differs in chemical composition; hence, it is essential to study the effect of material anisotropy. Furthermore, the effects of corner radius-to-thickness ratios on the residual stress through-thickness distribution were examined for each case. 

## 2. Numerical Simulation of Press-Braked Operation

### 2.1. General Methodology

**Figure 2:**
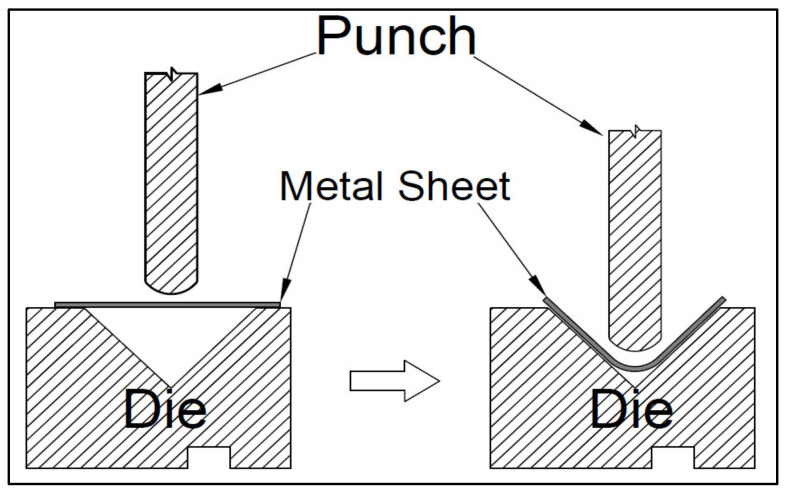
Press-braking operation.

Press-braking is a metal forming process performed at room temperature. This process occurs by folding a flat metal sheet along the member length using a punch and die, as shown in Figure 2. The aim of this study was to employ a 3D-FE modelling technique using the finite element method in ABAQUS software [27], in order to predict the residual stresses induced by the press-braked process in stainless steel sections. The investigation focused at the corner zone as the stainless steel thin plate experiences high bending force with the bent angle can reach up to 90° because of the punch movement. The stresses produced in the coiling/uncoiling and the flattening processes (i.e., initial stresses) were considered for the purpose of comparing the 2D-FE in [14,17] and 3D-FE adopted in this study. However, these initial stresses were not considered in the parametric study and thus the effect of the coil radius was neglected. Parametric study predicted the residual stress induced from the press-braking only. 

The simulation of press-braking covers two steps (forming and springback). Many studies preferred the dynamic explicit method over the implicit method in the sheet metal forming stage [17,24,25,28,29,30]. One advantage of the explicit algorithm is resolving complicated contact problems and convergence with improved efficiency. Additionally, it requires less system resources in large models compared to the implicit method. Yet, implicit has the advantage of handling the springback calculation more efficiently. Thus, Finn et al. [28] proposed a coupled method to exploit both algorithms advantages. Their approach used an explicit method to simulate the forming process while the implicit method used for springback simulation.

**Figure 3:**
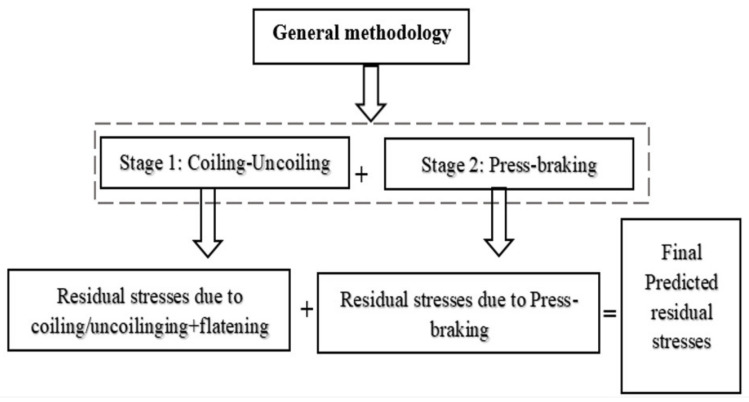
General Methodology.

As mentioned earlier, this study took into account the stresses produced from coiling/uncoiling-flattening processes for comparison purposes. Figure 3 shows a general methodology to predict the residual stresses. Stage 1 is essential for comparison with the 2D-FE technique in [14] where previous studies [12,31] demonstrated the effect of coil radius-to-thickness ratios at D/t < 800.

Figure 3 shows the general methodology of the finite element modelling strategies implemented in this work. The first step is the coiling-uncoiling stage then proceed to the press-braking stage and to finally predict the residual stresses. In the coiling-uncoiling stage, the residual stresses are first induced due to the winding process during packaging followed by the uncoiling and flattening process during the material preparation stage for press-braking. These residual stresses are carried on to the press-braking stage in which induces additional residual stresses. The next section describes the steps implemented to produce and configure the model to predict the residual stresses.

### 2.2. Modelling Steps

#### 2.2.1. Geometrical Properties

**Figure 4:**
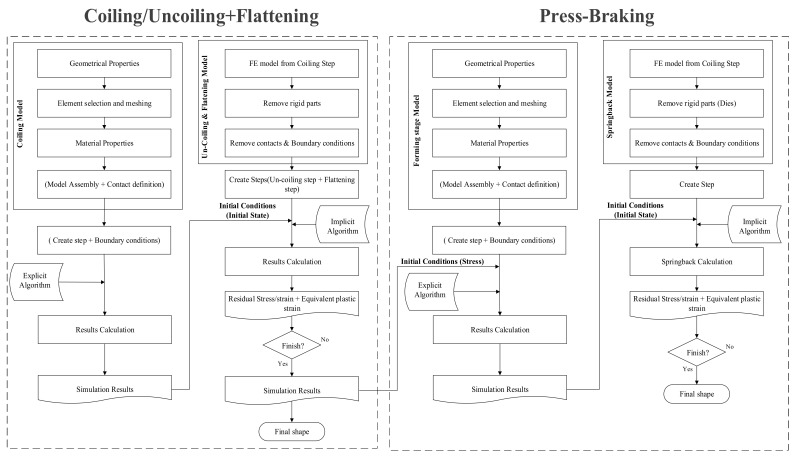
Flowchart of the modelling and simulation process.

**Figure 5:**
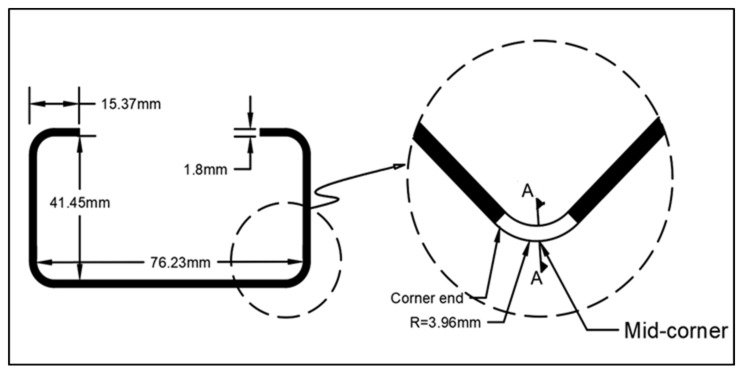
Geometry aspects of PBC14.

Figure 4 shows the detailed process implemented in the finite element simulation. Both modelling stages contain three parts: one deformable steel sheet and two rigid analytical parts. This research was meant to form only one corner. Mutafi et al. [24] demonstrated that the residual stress distribution did not change significantly at 4 mm from the edge. Therefore, the sheet dimensions were 100 mm (width) × 50 mm (length) to predict the residual stresses raised from the formed corner. This study considered the geometrical aspects of section PBC14 tested in [1] for comparison purposes as Quach et al. [14] employed 2D-FE in their work. Figure 5 shows the geometrical aspect of PBC 14.

**Figure 6:**
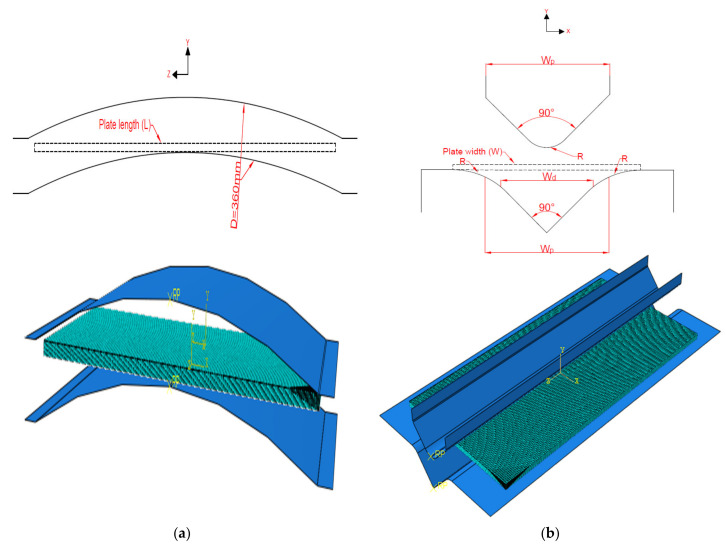
Models sketch and configuration: (**a**) coiling/uncoiling + flattening (**b**) press-braking.

The rigid parts (die and punch) in stage 1 were used in the forming process to gain the coiling curvature (*D/t* = 200). Figure 6a shows the dimensions and model configuration of stage 1. In stage 2, to form 90° angle, the basic requirements for die opening and punch size must be considered using the following equations (all notations are defined at the end of the paper):

(1) $$W_{d}\geq \sqrt{2}\left(R+t\right)$$

The punch size *W_p_* should be considered as follows:

(2) $$W_{p}=W_{d}+2R\left(1-cos45^{{}^{\circ}}\right)$$

This study employed the 3D-FE technique and compared it with 2D-FE technique performed by Quach et al. [12], thus similar dimensions for punch and die which was explained in [32] was used. The die opening was taken as $W_{d}=2.5\left(R+t\right)$ as shown in Figure 6b.

#### 2.2.2. Element Selection and Meshing

For both stages, the dies and punches were modelled as the rigid analytical parts. The steel sheet was modelled as a deformable continuum (solid) element “C3D8R”. The C3D8R element is an 8-node linear brick, with reduced integration and hourglass control [27]. Studies showed that continuum (solid) elements are the most appropriate to describe the deformation in the formed sheet metal through-thickness. The shell elements are not a good option for simulations where plastic deformation through thickness is important especially for plate metal forming process with small corner radius [18,33].

Medium mesh size (i.e. 240,000 solid elements) was selected in this study as in [24] proved to provide the optimal results. The size of a single element is approximated as 0.333 W × 1.0 L × 0.113 D. 

#### 2.2.3. Material Properties

##### Description of Stress-Strain Behaviour

In [13,14], the material properties for stainless steel were extracted from the existing literature [34]. UNS31803 duplex alloy was implemented to investigate the residual stresses using the 2D-FE technique. Therefore, to establish a comparable evaluation between the 2D-FE and 3D-FE techniques, the same material properties were adopted in this study. 

Equation (3) is used to approximate the through-thickness of σ_0N_ from σ_0D_. Defining the material anisotropy of UNS31803 in the FEM requires the value of σ_0N_ through the plate thickness, which is not readily available in the literature:

(3) $$\sigma_{0N}≅\sigma_{0D}≅\sqrt{3}\tau_{OXZ}$$

A compression coupon test in transverse, longitudinal and diagonal directions was conducted in [34] to obtain the mechanical properties of UNS 31803 duplex as shown in Table 1. The Poisson’s ratio assumption was set to be 0.3 for ν_xz_ (or ν_31_). The material modelling of the duplex stainless steel is further explained in the following two subsections.

##### Nonlinear Strain Hardening

In finite element analysis (FEA), the uniaxial longitudinal compression (LC) stress-strain curve was implemented to define the hardening behavior. The three-stage full-range stress-strain model, Equation (4), was implemented to define the relationship of the nominal stress-strain across the entire range of compressive strains in a longitudinal direction. It was considered that the strains due to coiling from large curvatures can significantly exceed the complete strain at 0.2% proof stress. This relationship was proposed by Quach et al. [35,36]:

(4) $$\varepsilon =\left\{\begin{array}{c} \frac{\sigma }{E_{0}}+0.002{\left(\frac{\sigma }{\sigma_{0.2}}\right)}^{n},\;\sigma \leq \sigma_{0.2}\\ \frac{\sigma -\sigma_{0.2}}{E_{0}}+\left[0.008+\left(\sigma_{0.1}-\sigma_{0.2}\right)\left(\frac{1}{E_{0}}-\frac{1}{E_{0.2}}\right)\right]\\ \times {\left(\frac{\sigma -\sigma_{0.2}}{\sigma_{1.0}-\sigma_{0.2}}\right)}^{n_{0.2,\;1.0}^{\prime }}+\varepsilon_{0.2},\;\;\;\sigma_{0.2}<\sigma \leq \sigma_{2.0}\\ \frac{\sigma -a}{b\mp \sigma },\;\sigma >\sigma_{2.0} \end{array}\right.$$

The basic Ramberg-Osgood parameters can be found in design codes, such as [37,38]. To model the nonlinear hardening behavior of anisotropic metals, a few steps are required as an approach by means of the input data in order for ABAQUS [27] to accept it. The relationship between $\bar{\sigma }$ and $\bar{\varepsilon }$_p_ is the final definition which needs to be introduced as the input data [27]. The true stress $\sigma_{t}$ and the true plastic strain $\varepsilon_{tp}$ are first converted from the nominal stress-strain data for the longitudinal compression (LC) (Equations (5)–(7). They are defined by the three-stage full-range stress-strain model (Equation (4)), using the following equations [39]:

(5) $$\sigma_{t}=\sigma_{n}\left(1\pm \varepsilon_{n}\right)$$

(6) $$\varepsilon_{t}=\pm \ln\left(1\pm \varepsilon_{n}\right)$$

(7) $$\varepsilon_{tp}=\varepsilon_{t}-\frac{\sigma_{t}}{E_{0}}$$

Note: positive sign corresponds to tension, minus sign corresponds to compression. To convert the true stress $\sigma_{t}$ and true plastic strain $\varepsilon_{tp}$, anisotropy parameters must be defined first [40]:

(8) $$F_{0}=\frac{1}{2}\left[\frac{1}{\sigma_{0N}^{2}}+\frac{1}{\sigma_{0L}^{2}}-\frac{1}{\sigma_{0T}^{2}}\right]$$

(9) $$G_{0}=\frac{1}{2}\left[\frac{1}{\sigma_{0T}^{2}}+\frac{1}{\sigma_{0L}^{2}}-\frac{1}{\sigma_{0N}^{2}}\right]$$

(10) $$H_{0}=\frac{1}{2}\left[\frac{1}{\sigma_{0N}^{2}}+\frac{1}{\sigma_{0N}^{2}}-\frac{1}{\sigma_{0L}^{2}}\right]$$

The through-thickness 0.2% proof stress, $\sigma_{0N}$, is approximated in Equation (3) and Table 1. Finally, the equivalent true stress $\bar{\sigma }$ and the equivalent true plastic strain $\bar{\varepsilon }$_p_ for longitudinal compression (LC) are estimated using the following expression [40]:

(11) $$\bar{\sigma }=\sqrt{\frac{3}{2}\left(\frac{F_{0}+G_{0}}{F_{0}+G_{0}+H_{o}}\right)}\sigma_{t}$$

(12) $$\bar{\varepsilon }p=\sqrt{\frac{2}{3}\left(\frac{F_{0}+G_{0}+H_{o}}{F_{0}+G_{0}}\right)}\varepsilon_{tp}$$

**Figure 7:**
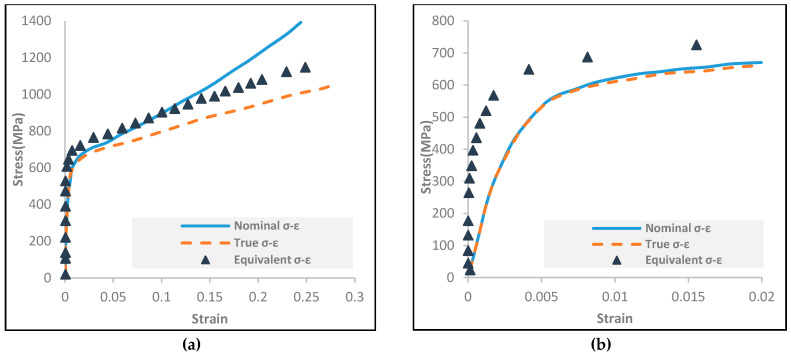
Stress-strain curves for longitudinal compression UNS31803 duplex stainless steel (**a**) Full stress-strain curve; (**b**) Initial stress-strain curve.

Figure 7 shows curves of nominal stress-strain, true stress-strain, and the equivalent stress-equivalent plastic strain for the longitudinal compression. 

##### Material Anisotropy 

A local coordinate system was employed to define the material anisotropy in the FE model in order to describe every element material direction, which corresponds with the global coordinate system. During the finite element simulation, each element material direction followed the rotation of the material as it deformed. 

The material model of orthotropic elasticity and the anisotropic metal plasticity describe the material anisotropy in FEA. The engineering constants in Equations (14)–(17) define the orthotropic elasticity model. E_3_ and E_1_ demonstrate the initial elastic moduli of E_0z_ and E_0x_ for the longitudinal compression (LC) and transverse compression (TC), respectively. E_2_ is the initial elastic modulus in the through-thickness direction “E_0y_”, which is not available in the literature. Therefore, an assumption was made regarding the fact that E_2_ has a similar value as the initial elastic modulus E_3_ (i.e., E_0z_). The Poisson’s ratios assumed to be 0.3 for ν_12_ and ν_23_. Also, ν_31_, was assumed to be 0.3, leading to a value of 0.26 for ν_13_, as stated by:

ν_xz_ = ν_zx_E_0z_/E_0x_ (13)

The shear moduli were considered as the initial shear elastic moduli of a UNS 31803 duplex stainless steel given in Appendix B of the AS/NZS 4673 Standard [37]. It should be mentioned that the values of the Poisson’s ratio and shear moduli (in local coordinate system) are insignificant. However, they have been allocated sensible values based on the data from different sources. To summarize, the values of the engineering constants are as follows:

E_3_ = E_0z_ = 181.65 GPa; E_2_ = E_0y_ = 210.00 GPa (14)

E_1_ = E_0x_ = 210.00 GPa (15)

ν_12_ = ν_23_ = 0.30; ν_13_ = ν_31_E_1_/E_3_ = 0.26; (16)

G_12_ = G_13_ = G_23_ = 75.00 GPa; (17)

The anisotropic metal plasticity model in ABAQUS is defined by Hill’s yield criterion for anisotropic materials and isotropic hardening flow rule. While the nonlinear strain hardening is modeled by specifying a stress-strain curve of “reference’’, six yield stress ratios, R_ij_ which define the state of plastic anisotropy in ABAQUS. The six yield stress ratios are defined as:

(18) $$R_{11}=\frac{\sigma_{0,11}}{\sigma_{0}};\;R_{22}=\frac{\sigma_{0,22}}{\sigma_{0}};R_{33}=\frac{\sigma_{0,33}}{\sigma_{0}};$$

(19) $$R_{12}=\frac{\tau_{0,12}}{\tau_{0}};R_{13}=\frac{\tau_{0,13}}{\tau_{0}};R_{23}=\frac{\tau_{0,23}}{\tau_{0}};$$

where:

(20) $$\tau_{0}=\sigma_{0}/\surd 3$$

The initial yield stresses, σ_0,ii_, were considered as compressive 0.2% proof stress in the three principal directions (Table 1), in which the through-thickness 0.2% proof stress σ_0N_ (i.e., σ_0,22_) is approximated by the diagonal 0.2% proof stress σ_0D_ Equation (3). To be precise:

σ_0,11_ = σ_0T_ = 617.0 MPa; σ_0,22_ = σ_0N_ = 610.0 MPa; σ_0,33_ = σ_0L_ = 527.0 MPa. (21)

The values of shear yield stresses τ_0,ij_ are insignificant for this plane strain bending problem. Nevertheless, the shear yield stress τ_0,13_, which is τ_0XZ_, is approximated by $\sigma_{0}/\surd 3$ (see Equation (1)). Both the τ_0,12_ and τ_0,23_ are taken as σ_0_ such that R_12_ = 1 and R_23_ = 1. To be specific:

τ_0,12_ = τ_0_; τ_0,13_ = τ_0XZ_ = 610.0/√3 MPa; τ_0,23_ = τ_0_ (22)

This study considered the stress-strain curve for longitudinal compression (LC) in order to describe the stainless steel hardening behavior. Hence, after converting the stress-strain curve for longitudinal compression (LC) to the equivalent stress-equivalent plastic strain, the data was used to define the “reference” stress-strain curve. As the reference yield stress σ_0_ is the yield stress of the “reference” stress-strain curve, the reference yield stress σ_0_ was considered as the equivalent stress $\bar{\sigma }$ converted from the compressive 0.2% proof stress σ_0L_ in the longitudinal direction:

(23) $$\sigma_{0}=\sqrt{\frac{3}{2}\left(\frac{F_{0}+G_{0}}{F_{0}+G_{0}+H_{o}}\right)}\sigma_{0L}$$

##### Material Properties for Parametric Study 

This parametric study considered four types of stainless steel available in the market (ferritic, austenitic, duplex, and lean duplex). These types differ from each other based on their chemical composition and percentage of the base material compositions. Nickel content is the main parameter that differentiates them. Besides, the nickel content governs the type price. Ferritic stainless steel is the only type that has 0% of nickel content, thus making it the most economically competitive, and potentially have the most comprehensive application in the building industry [41,42]. It could be the most appropriate and economical type to be used in construction. Austenitic and duplex have 8–10% of nickel content. Lean duplex is a new type of stainless steel with a low content of nickel (around 1.5%) and may offer improved economic benefits over existing grades. The material referred to as “lean duplex stainless steel” possesses higher strength than the austenitic grades and is less expensive. Table 2 summarises the material properties for each type.

As mentioned in Section 2.2, the three-stage full-range stress-strain model Equation (4) was used to describe the nominal stress-strain relationship across the entire range of strains in the longitudinal direction. The nominal stress-strain data was converted using Equations (5)–(7) to define the $\sigma_{t}$ and $\varepsilon_{tp}$. To define $\bar{\sigma }$ and $\bar{\varepsilon }$_p_ from Equations (11) and (12), the definition of anisotropy parameters is required (Equations (8)–(10)). Since the diagonal initial stress is not available in the literature (Ferritic [43,46], and Lean Duplex [45]), Equation (3) is not applicable to estimate the through-thickness initial yield stress σ_0N_. It was assumed that $\sigma_{0N}≅\sigma_{0T}$. Table 3 summarises the material anisotropy.

#### 2.2.4. Assembly and Contact Definition

The model parts were assembled in each stage and face-to-face constraints were employed to position the model in the correct place. These constraints were between the rigid part and the deformable part. The first constraint was between the die surface and the outer surface of the stainless steel sheet with no clearance distance to allow for full contact between the constrained surfaces. The second constraint was between the punch and the outer surface. Also, the clearance between them was zero to ensure full contact between the two parts.

The contact pair between the parts were modeled as surface-to-surface interactions where rigid parts selected as first surface and the stainless steel sheet was the second surface with finite sliding formulation. For the contact interaction property, it was modeled as hard contact as shown in Figure 6.

#### 2.2.5. Create step and Boundary Conditions

##### Forming (Explicit)

Estimating the optimal step time for the forming step is essential to ensure reliable results. ABAQUS [27] generally recommends the punch speed to be less than 1% of the steel wave speed (5000 m/s). Moreover, the manual suggested that the punch speed to have 1 m/s or higher for typical forming process. However, the punch moved distance in both stages was 0.07 m as shown in Figure 6. Thus, 0.07 m/s was chosen as the speed equivalent to the recommended speed, which lead to the estimated step time of 0.07 s. In several trials, the optimal first step time of 0.1 s was selected with result showed some localized damage and kinetic energy higher than 10% of internal energy when the mass scaling of 350 was used in order to speed up the analysis [27,47].

For boundary conditions, the die was restricted from moving in all directions by applying fixed boundary conditions at its reference point (RP). Displacement loading was applied to the punch in the y-direction only in order to form the steel sheet. For stage 2 (press-braking), stresses obtained from stage 1 were considered as the initial condition by employing the predefined fields (stress).

##### Springback

For springback analysis in both stages, it is essential to remove the rigid parts, contacts, and explicit step but keeping the deformable part only. Before creating the implicit step, the results from the forming step (Explicit) were taken as the initial condition for the next simulation employing a predefined field (initial state) in the program. Stage 1 contained two steps, the first implicit step (general static) created to simulate the uncoiling process. The steel sheet was set to be free of any boundary restrictions. The second implicit step (general static) was for the flattening process. The boundary condition in this step was restricted in the normal direction (y-direction) in order to flatten the steel sheet. Stage 2 had only one step similar to the first step in stage 1.

## 3. Results Comparison

### 3.1. Model Verification

The numerical results were compared with the results of the empirical formula used for predicting the transverse residual strain (ε_x_). Cook (1966) [48] mentioned that plastic bending operation would have 5% shift in the neutral axis towards the compressive face. Taking this into account, the prediction for ε_x_ can be modified as follows:

(24) $$\varepsilon_{x}=\frac{-y+0.05t}{R+0.45t}$$

Another empirical formula, presented by Johnson and Mellor [49] for bend angles exceeding 70° and for width to thickness ratios of more than 10, considers the shifting in the neutral axis around 5% towards the compressive face. The equation is as follows:

(25) $$\varepsilon_{x}=\frac{-y+0.05t}{R+0.55t}$$

This research took these equations for model verification. In order to ensure accuracy, two transverse lines were selected (z = 0 mm and 50 mm) where z = 0 mm represents ε_x_ result in the corner edge region and z = 50 mm represents the mid-corner region.

**Figure 8:**
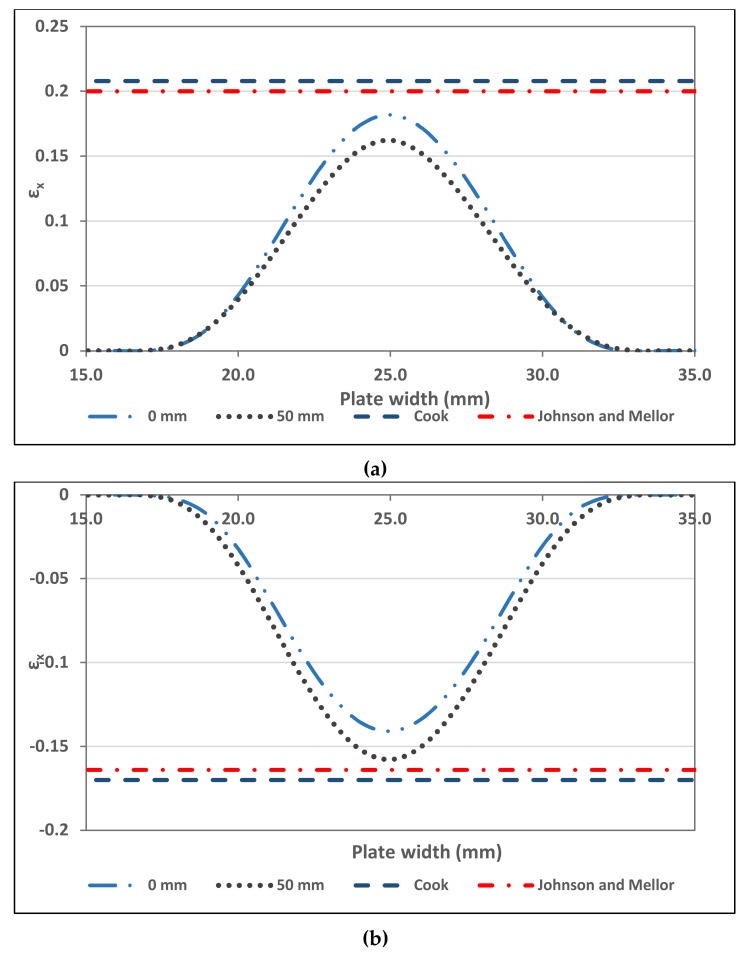
Transverse residual strain: (**a**) Outer surface (**b**) Inner surface.

Figure 8 shows the comparison of the ε_x_ values obtained from 3D-FE analysis and the analytical predictions using Cook [48] Equation (14) and Johnson and Mellor [49] Equation (15). The peak transverse residual strain was linear along the corner area, as the analytical predictions presume. Furthermore, the numerical findings at each point on the plate approximated the strains.

Figure 8 shows higher transverse residual strain ε_x_ at the edge zone (z = 0 mm) than the mid-corner (z = 50 mm) in the outer surface. On the other hand, ε_x_ at the edge zone (z = 0 mm) is lower than the mid-corner (z = 50 mm) in the inner surface. This differences are due to the Poisson’s effect, where formed corners tend to bend at the edge due to the lack of transverse restraint [25]. As the outer surface in compressive mode and the inner surface in tensile mode, hence, the edge zone exhibited different strains than the mid-corner zone.

The comparison between the 3D-FE results and the empirical formula showed a close agreement for z = 0 mm and 50 mm with 91–78% differences as shown in Figure 8a. In Figure 8b, the comparison showed close agreement with 96–83% differences. Therefore, this agreement shows that the finite element modeling strategies can simulate the press-braking process for stainless steel sheet effectively.

### 3.2. Residual Stress Distribution

This section describes the residual stresses distribution in the formed corner section. The distribution along the bending region for the inner and outer-surfaces is explained.

#### 3.2.1. Transverse Residual Stresses

**Figure 9:**
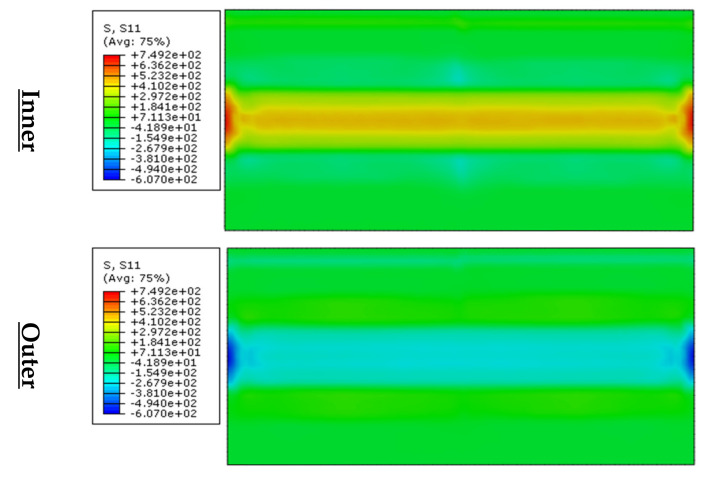
Transverse residual stresses distribution at bending region (Unreformed shape).

The distribution of transverse residual stresses (S11) as shown in Figure 9 shows that the inner-surface contains tensile-stresses. These stresses reach their peak at the corner edge. The tensile-stresses seem to have the same distribution when they are far from the edge and closer towards the mid-plate. Far from the corner edges, the stress gradually decreases towards the corner end where it can be noticed the existence of compressive stress. This dramatic change in stresses from tensile to compressive was discussed by Mutafi et al. [24] where the flat region beyond the corner does not obey similar stress distribution.

Compressive-stresses cover the outer corner region. Similar to the inner zone, compressive-stresses reach its peak at the corner edge. However, the mid-corner zone has compressive-stresses, which are lower than that of the corner edge. Far from the corner edge, the stress distribution at the corner zone seems steady from mid-corner toward the corner ends.

#### 3.2.2. Longitudinal Residual Stresses

**Figure 10:**
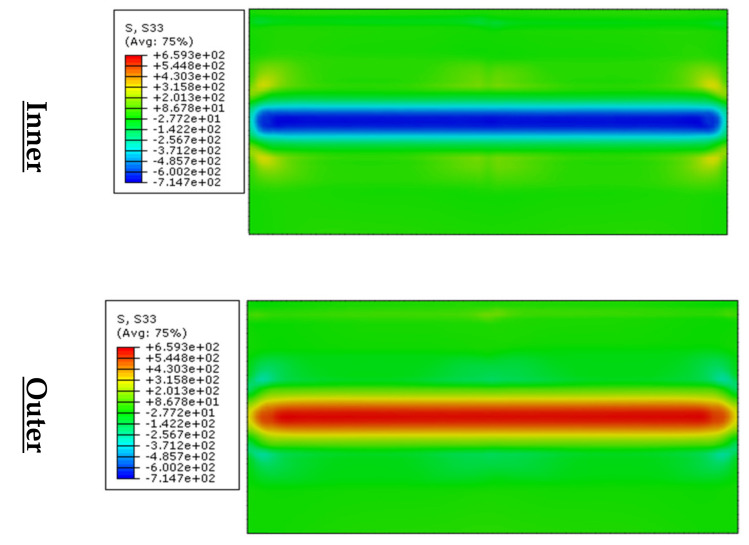
Longitudinal residual stresses distribution at corner region (Unreformed shape).

The distribution of the longitudinal residual stresses (S33) shows that inner-surface contains compressive-stresses, as shown in Figure 10. 

These stresses reach their peak near the mid-plate and decrease near the corner edge. The compressive-stresses seem to have the same distribution far from the edge towards the mid-plate. Compressive-stresses are higher near the mid-corner. At the corner, the tensile-stresses appear to take place near the edge. The existence of these stresses can be explained by the die/sheet interaction effect [12]. Tensile-stresses cover the outer corner region. Similar to the inner zone, tensile-stresses approach their highest point far from the edge. In addition, the mid-corner zone has tensile-stresses, which is higher than that of the corner end. Compressive-stresses exist near the corner edge and end due to the die/sheet interaction effect.

### 3.3. Residual Stresses Through-Thickness Distribution of Coiling-Uncoiling Process

This section explains the through-thickness distribution of residual stresses for coiling-uncoiling process at the cross-section A-A (mid-corner). The sub section will demonstrate the stresses variation for the uncoiling and flattening process. Individual nodes were picked from the mid-corner to explore the behaviour of residual stresses along the corner region. The selected nodes represent four different zones in the corner region. These zones are the edge (0 mm), near edge (4 mm), far edge (7 mm) and mid-section (50 mm).

#### 3.3.1. Transverse Through-Thickness Residual Stresses

**Figure 11:**
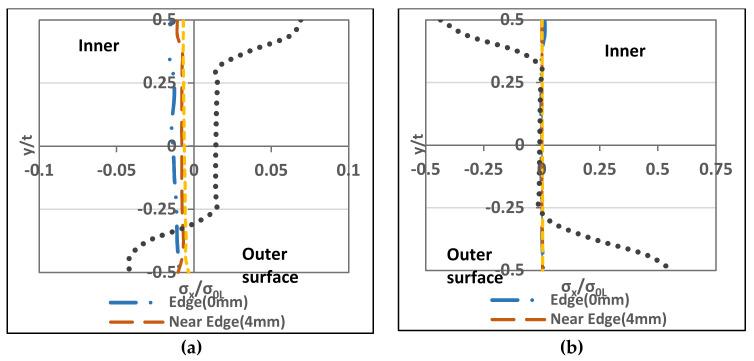
Through-thickness residual stresses’ variation due to uncoiling (**a**) Transverse stress; (**b**) Longitudinal stress.

Figure 11a shows the variation of through-thickness transverse residual stresses along the longitudinal direction of the final shape produced from stage 1. As explained in Section 3.2, the inner-surface contained tensile-stresses, whereas the outer zone contained compressive-stresses. The through-thickness distribution shows that the edge (0 mm), near edge (4 mm) and far edge (7 mm) contain compressive stresses from the inner surface to the outer surface and that they approach zero. However, the mid-section (50 mm) distribution is in tensile stress at the inner surface and compressive stress at the outer surface. Its peak tensile and compressive stresses exist at the inner and outer surfaces, respectively. The magnitude of the peak stress is small; therefore, the effect of coiling-uncoiling process can be ignored with regards to the transverse residual stress for metal forming technique (e.g., press-braking and roll-forming).

#### 3.3.2. Longitudinal Through-Thickness Residual Stresses

Figure 11b shows the variation of through-thickness longitudinal residual stresses along the longitudinal direction of the final shape produced from stage 1. As explained previously in Section 3.2, the inner-surface contained compressive-stresses, whereas the outer surface contained tensile-stresses. However, the through-thickness distribution shows that the edge (0 mm), near edge (4 mm) and far edge (7 mm) contain stresses which are close zero. 

In the mid-section (50 mm) the magnitude of the stress distribution exceed 0.5 σ_0L_ at the outer surface, which is tensile stress and approach 0.5 σ_0L_ at the inner zone, which is compressive. Thus, coiling-uncoiling effect cannot be ignored for metal forming technique particularly for large coil curvature.

### 3.4. Residual Stresses through-Thickness Distribution of Press-Braking

This section shows the through-thickness distribution of residual stresses of press-braking at cross-section A-A (mid-corner). The following sub section will demonstrate the stress distribution for the press-braking operation at the forming stage and springback stage. The same selected nodes discussed in Section 3.3 will be presented in this section for the through-thickness variation.

#### 3.4.1. Transverse Through-Thickness Residual Stresses

**Figure 12:**
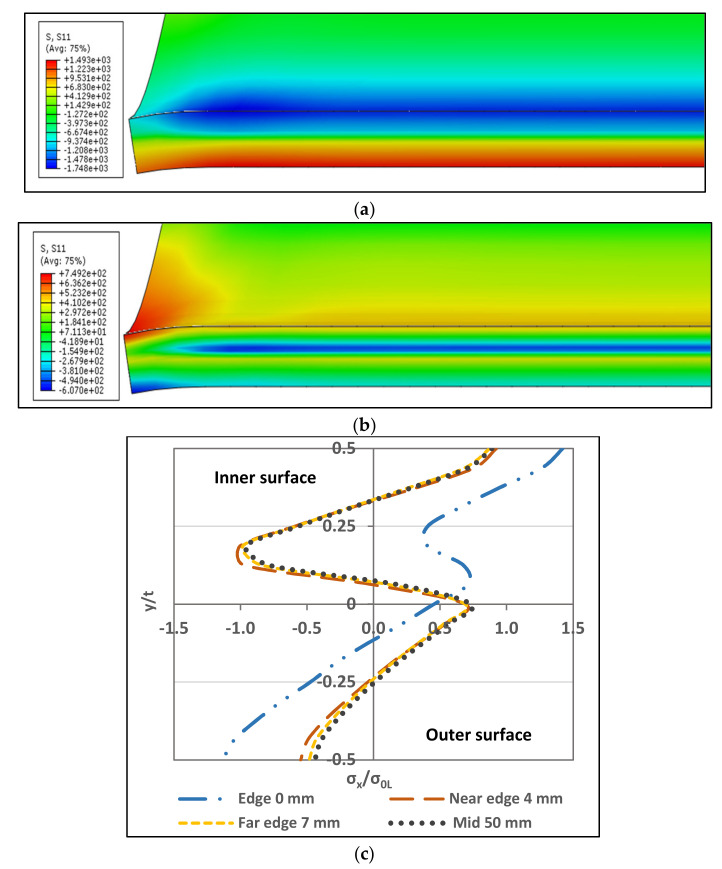
Transvers residual stresses distribution (A-A cross section view): (**a**) Stamping, (**b**) Springback (**c**) through-thickness variation.

Transverse through-thickness residual stresses show a dramatic change from forming to springback, as shown in Figure 12. 

During forming, the inner zone seems to be governed by compressive-stresses, whereas tensile-stresses control the outer zone. However, during springback, tensile-stresses exist in the inner zone, and compressive-stresses are in the outer part. At the forming stage, residual stresses vary uniformly from high compressive-stresses at the inner zone to high tensile-stresses at the outer zone. Furthermore, the mid-surface contains a combination of compressive-stresses (towards inner-surface) and tensile-stresses (towards outer-surface). In the springback stage, non-uniform residual stresses vary in which the inner parts contain tensile. The mid-surface (towards the inner part) has compressive-stresses. The mid-surface (towards the outer part) has tensile-stresses. Compressive-stresses cover the outer-surface. The area close to the edge zone does not show significant change during the stamping stage but it does for the springback stage as the tensile-stresses govern the mid-surface. 

Figure 12c shows the variation of through-thickness transverse residual stresses along the corner zone of the final shape produced from stage 2. As explained in Section 3.2, the inner-surface contained tensile-stresses, whereas the outer zone contained compressive-stresses. Through-thickness distribution at the edge (0 mm) illustrates that tensile-stresses approach its peak at the inner-surface. Compressive-stresses are at its highest magnitude on the outer-surface. Although, the rest of the selected lines show similar through-thickness variation from the edge location, the maximum values differ from the edge. The maximum tensile-stresses are at the inner-surface. Unlike the edge line, the mid-surface (towards inner-surface) contains peak compressive-stresses. Figure 10c shows that the maximum residual stresses (tensile and compressive) exist at the upper part (towards inner-surface). Carbon steel studied by Mutafi, et al. [24] showed that the transverse residual stress variation along the corner region has similar peak location (near mid-surface). However, stainless steel shows different variations at the edge where the peak tensile and compressive stresses exist at the inner and outer surfaces, respectively. Another point to compare is with the carbon steel in [24], for the stainless steel the peak tensile stress exists in the inner surface whereas for the carbon steel the peak tensile stress exists closer to the mid-surface. It is worth to mention that the residual stress in the transverse direction exceeds the yield limit at the edge only while the other regions the stresses maintain below the plastic limit. This, perhaps, is due to the effect of coiling-uncoiling process.

#### 3.4.2. Longitudinal Through-Thickness Residual Stresses

**Figure 13:**
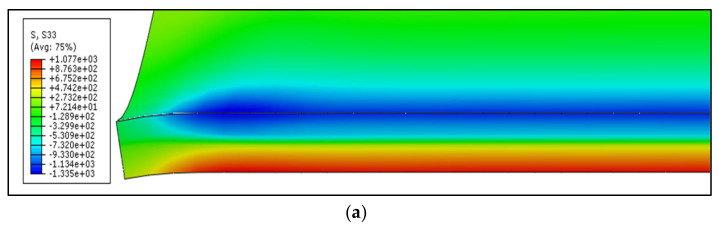

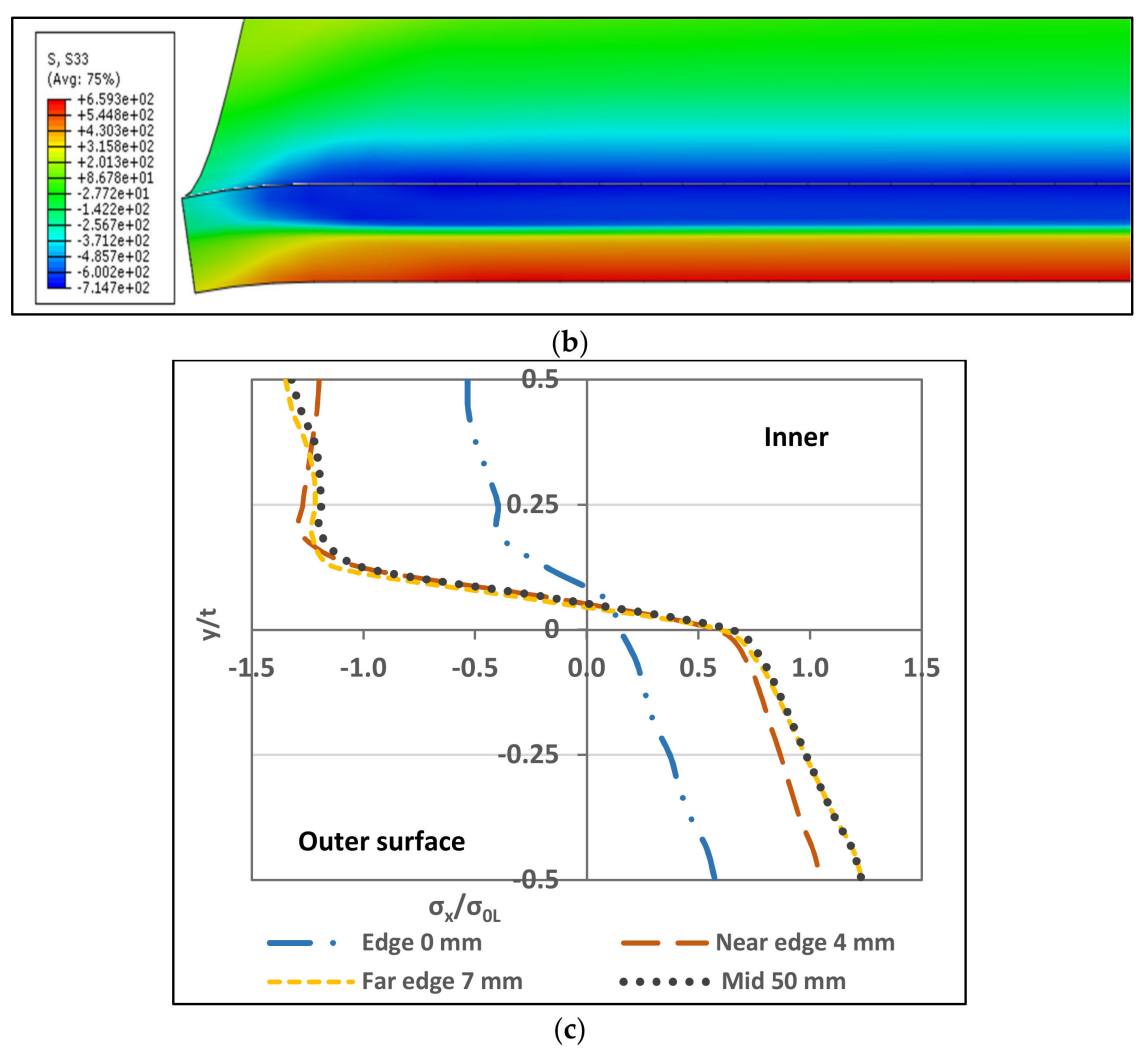
Longitudinal residual stresses distribution (A-A cross section): (**a**) Stamping, (**b**) Springback (**c**) Through-thickness variation.

Longitudinal through-thickness residual stresses show a similar variation from forming to springback, as shown in Figure 13. During stamping, the inner zone seems to be governed by compressive-stresses, whereas tensile-stresses govern the outer zone. Likewise, during springback, the compressive-stresses exist in the inner zone, and the tensile-stresses are in the outer part. At the forming stage, residual stresses vary uniformly from high compressive-stresses at the inner zone to high tensile-stresses at the outer zone. Furthermore, the mid-surface contains a combination of compressive-stresses (towards inner-surface) and tensile-stresses (towards outer-surface). At the springback stage, residual stress variations did not change; thus, the inner part contains compressive-stresses. The mid-surface (towards the inner part) contains compressive-stresses. The mid-surface (towards the outer part) has tensile-stresses, and tensile-stresses cover the outer-surface. The edge area shows a significant change in the modee of stresses during the stamping stage and springback stage.

Figure 13c shows longitudinal residual stresses through-thickness variation along the corner region of the final shape produced from stage 2. As explained previously in Section 3.2, the inner-surface contained the compressive-stresses, whereas the outer one contained the tensile-stresses. All through-thickness distribution of the selected lines demonstrates that compressive-stresses exist at the inner while the outer-surfaces contain the tensile-stresses. Maximum compressive and tensile stresses exist at the inner and outer surfaces, respectively. The edge line (0 mm) shows lower compressive and tensile stresses than the rest of all selected lines. For the edge line (4 mm), the peak compressive is near the mid-layer towards the inner-surface while the tensile is at the mid-layer. At the far edge (7 mm) and the mid-section (50 mm) the stresses illustrate similar distributions. Although, the stress at the edge line (0 mm) shows lower stress distribution trend, unlike the carbon steel studied in [24] where the stress at the edge line differ from the rest of the corner zone. All selected lines have closer neutral axis shifting, which means that the residual stress distribution is not dramatically disturbed near the edge. Finally, the peak longitudinal residual stress goes beyond the yielding limit except for stresses at the edge line (0 mm). This excessive residual stress can be explained by the effect of the coiling-uncoiling process however for edge line (0 mm) the lower residual stress happens because of the “Poisson effect” which is the cause of stress-release phenomena.

### 3.5. Residual Stress through-Thickness Variation and Comparison With 2D-FE Results

**Figure 14:**
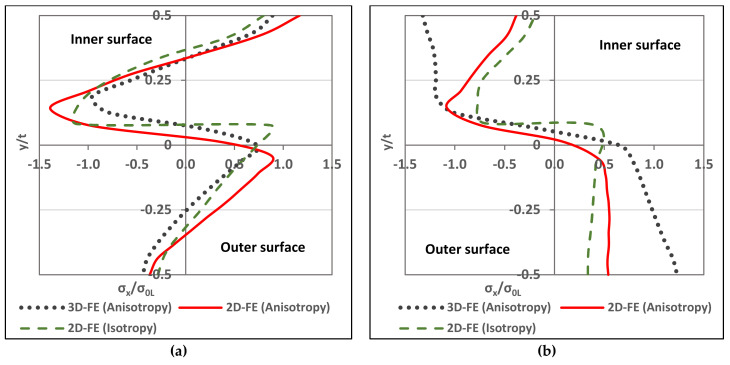
Through-thickness residual stresses’ variation comparison. (**a**) Transverse; (**b**) Longitudinal.

This section compares the through-thickness variation of the results obtained from Section 3.4 with 2D-FE results from [14,17]. For the 3D-FE technique, it was concluded by Mutafi et al. [24] that the through-thickness residual stresses have similar distribution far from the corner edge. In addition, they found that the 3D-FE technique has similar distribution with 2D-FE technique far from the corner edge. In this study, the effect of material anisotropy is the authors’ main concern with regards to the residual stress through-thickness variation. Quach et al. [14] considered material anisotropy in their 2D-FE analysis for the press-braked section as well as the stresses raised from the coiling-uncoiling process. However, Zheng, et al. [17] adopted the same material properties assuming it as isotropic material while neglecting the effect of the material anisotropy. Also, they assumed that the coiling-uncoiling effect is minor and can be ignored. The comparison between [17] and [14] showed differences in the distribution. The following section compare the results at z = 50 mm with results in [14,17]. Figure 14 illustrates the stress distribution and differences between:

1. 3D-FE (anisotropy): the results obtained in this study that considered the material anisotropy effect.
2. 2D-FE (anisotropy): the results extracted from [14] which considered material anisotropy effect.
3. 2D-FE (isotropy): the results extracted from [17] which considered material isotropy (No anisotropy effect).

#### 3.5.1. Transverse Stresses

In Figure 14a, the distribution shape of the three trends is the same as a zigzag distribution. Despite the similarities in the distribution shape, peak tensile stress form the 3D-FE and the 2D-FE (anisotropy) exist in the inner surface and the peak compressive stress located above the mid-surface. The 2D-FE (isotropy) peak compressive and tensile stress located above the mid-surface and the tensile stress is closer to the mid-surface. Meanwhile, the 3D-FE (anisotropy) do not yield at its peak stresses (normalized stress σ_x_/σ_0L_ > 1), while the 2D-FE (anisotropy) exceed the yielding limit in its peak stresses. The 2D-FE (isotropy) go beyond yielding limit in compressive stress but do not exceed it in tensile stress. It can be notice that there are differences between the 3D-FE (anisotropy) and the 2D-FE (anisotropy) models in terms of the stress peak amount and location despite due consideration was given to the material modeling of anisotropy with the same description of stress-strain behavior. This might be due to the material model in the transverse direction is ignored, which is the bending direction. Hence, the geometrical aspects and the choice of finite element technique have a dominant influence in the through-thickness stress distribution in the transverse direction. The 2D-FE techniques adopted the plane strain assumption and presumed linear stress in the through-thickness distribution along the corner zone. In contrast, the study showed that the stress distribution in through-thickness direction along the corner zone is not linear [23,24].

#### 3.5.2. Longitudinal Stresses

In Figure 14b, the distribution shape of the three trends have a few differences. Where the 3D-FE (anisotropy) has differences comparing with the other 2D-FE distributions. For the peak stresses, the 3D-FE (anisotropy) tensile and compressive stresses exist in the outer and inner surfaces, respectively. The maximum tensile stress for the 2D-FE (anisotropy) located at the outer surface while the compressive stress can be found above the mid-surface. On the other hand, the 2D-FE (isotropy) peak tensile and compressive stresses exist above the mid-surface whereby the tensile stress is closer to the mid-surface. The 2D-FE (isotropy) does not show any excessive yielding. Only the compressive stress has reach yielding for the 2D-FE (anisotropy). Nevertheless, the 3D-FE (anisotropy) yield in both tensile and compressive stresses at the outer and inner surfaces. The neutral axis shift differs for each technique, the 2D-FE (anisotropy) show the lowest shift while the 2D-FE (isotropy) has the highest. The effect of coiling-uncoiling process can be notice clearly in the 3D-FE (anisotropy) simulation results. This shows that implementing a full finite element procedure from the beginning of coiling-uncoiling process gives different outcomes when compared to the finite element simulation, which incorporated estimate of the residual stress due to coiling-uncoiling process using the analytical method. In terms of material effect, the 2D-FE (isotropy) show similar distribution trend with the 2D-FE (anisotropy). However, the 2D-FE (isotropy) stress outcomes are less than the 2D-FE (anisotropy). It is worth to mention that the 2D-FE (isotropy) presented in [17] ignored stresses from the coiling-uncoiling process and stated that their effect is minor. Thus, the 2D-FE (isotropy) shows the lowest stresses at the inner and outer surfaces. However, the peak stress located near the mid-surface is attributed to the isotropic assumption which smaller than that of using anisotropic assumption. In summary, considering material anisotropy is important to accurately predict residual stresses in the longitudinal direction.

## 4. Parametric Study

As explained in Section 1, this section investigates the residual stress distribution for the stainless steel types available in the market (ferritic, austenitic, duplex and lean duplex). The study selected three different corner radius-to-thickness ratios for each type (R/t = 2, 2.5, and 3). Material properties are summarised in Table 2 and Table 3. Mid-corner lines were selected for this study and the results gained from the study represented the four plate zones: edge (0 mm), near edge (4 mm), far edge (7 mm) and mid-section (50 mm). The finite element analysis for this study did not include the coiling-uncoiling process. It emphasis mainly on the through-thickness distribution due to press-braking for each type. Hence, only the press-braking stage (Stage 2) is considered in this investigation, see Figure 3.

### 4.1. Ferritic Stainless Steel

#### 4.1.1. Transverse

**Figure 15:**
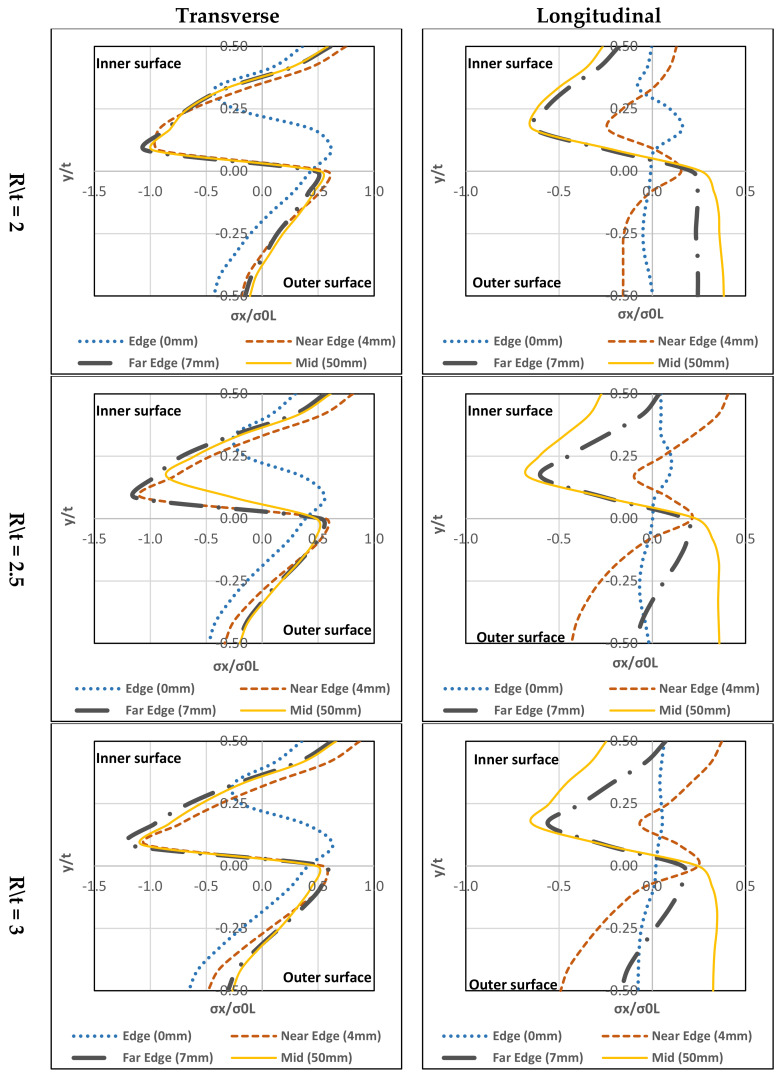
Through-thickness residual stresses distribution of ferritic stainless steel.

In Figure 15, it can be seen that all three (R/t) ratios have similar stress distribution forms. Maximum tensile residual stress exists at the inner-surface and maximum compressive-stress can be found at the mid-layer (towards the inner part). Except for the edge (0 mm), a small difference in the stress distribution with regards to the peak residual stress (compressive residual stress is far from the mid-layer and closer to the inner-surface) is observed. Tensile residual stress is on the upper mid-layer. For all R/t ratios, the highest compressive-stress located at the far edge (7 mm) and the highest tensile-stress located at the near the edge (4 mm).

#### 4.1.2. Longitudinal

In Figure 15, there are a few differences between the three R/t ratios. For R/t = 2, at the near edge (4 mm) the inner-surface has the tensile-stresses and the outer-surface has the compressive-stresses.

The others have compressive-stresses at the inner-surface and tensile-stresses at the outer-surface. Peak compressive-stress is located at the mid-layer towards the inner-surface while the peak tensile-stress is at the outer-surface. For R/t = 2.5 and 3, the stress distribution form is tensile-stresses at the inner-surface and compressive-stresses at the outer-surface except for the mid-section (50 mm), the compressive-stresses are at the inner-surface, and tensile-stresses are at the outer-surface. The tensile-stresses reach the highest level near the edge (4 mm) at the inner-surface. However, the mid-section (50 mm) has closer tensile-stresses at the outer-surface. For compressive-stresses reach a peak at the mid-layer towards the inner-surface.

### 4.2. Austenitic Stainless Steel

#### 4.2.1. Transverse

**Figure 16:**
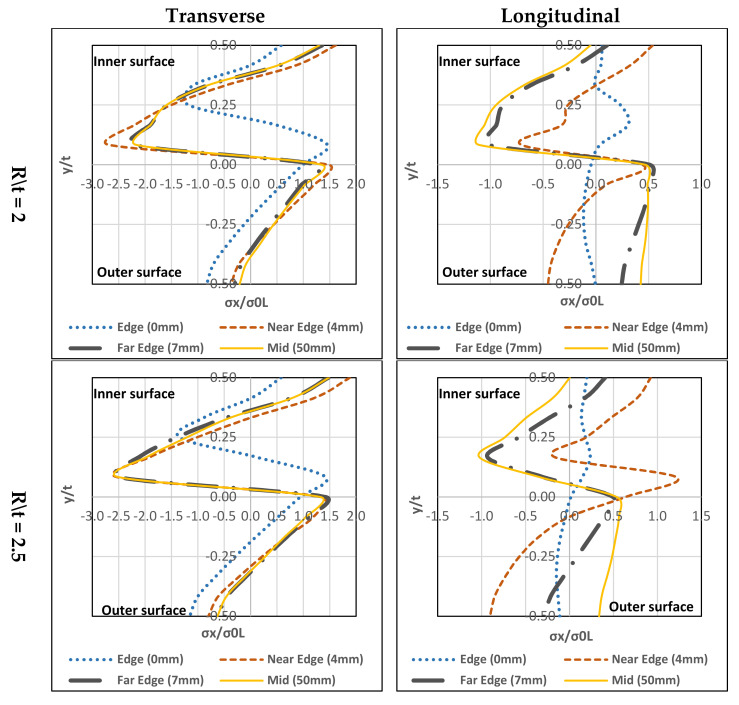

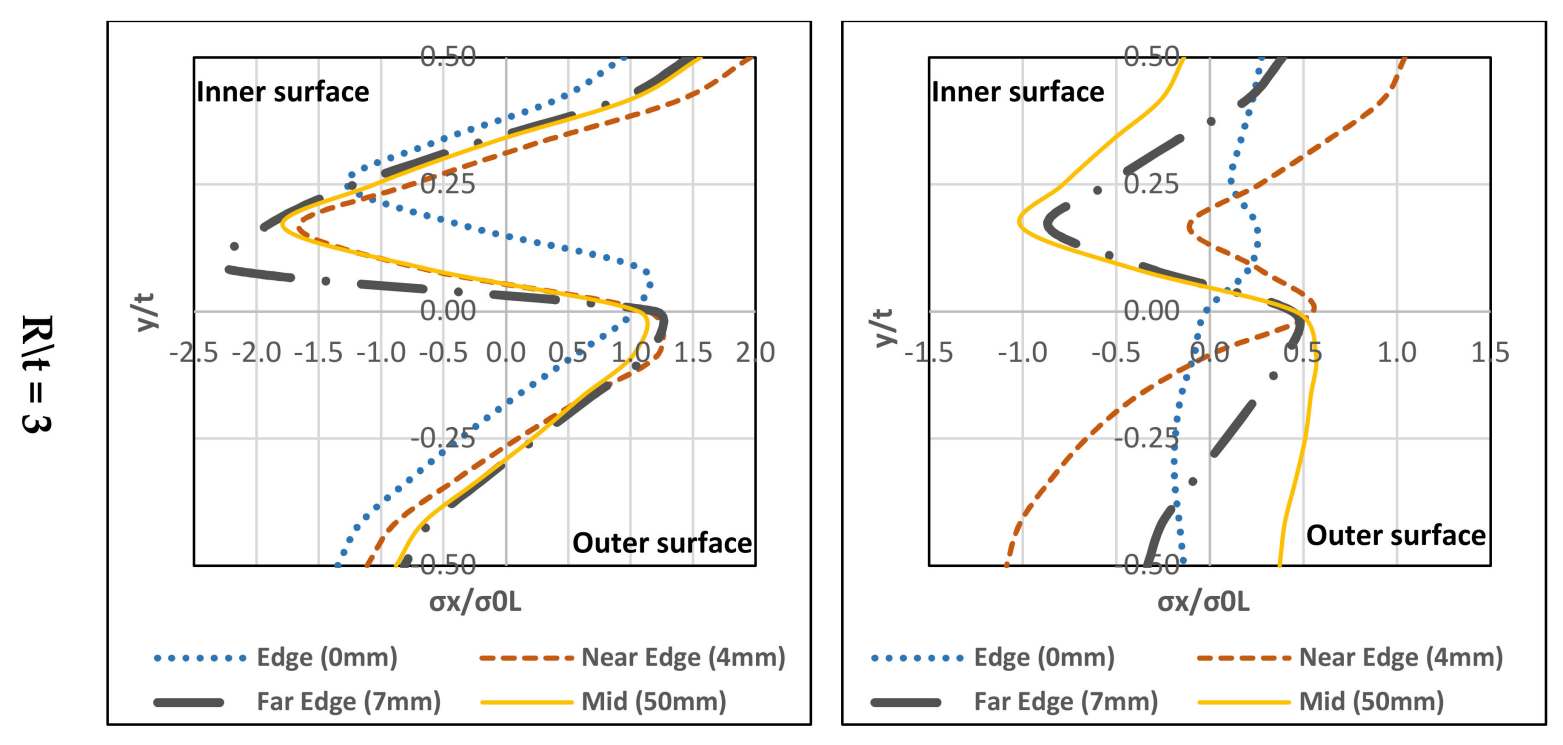
Through-thickness residual stresses distribution of austenitic stainless steel.

In Figure 16, the three (R/t) ratios have similar stress distribution forms. Location of the highest tensile-stress is at the inner-surface and highest compressive-stress is at the mid-layer (towards the inner). Except at the edge (0 mm), the trend differs slightly with regards to the peak residual stress (compressive residual stress is far from the mid-layer and closer to the inner-surface. Tensile residual stress is at the upper mid-layer).

#### 4.2.2. Longitudinal

In Figure 16, there are a few differences in terms of stress distribution between the three R/t ratios. For the edge (0 mm), near the edge (4 mm) and far edge (7 mm) the inner-surface have tensile-stresses and the outer-surface has compressive-stresses except for the far edge (7 mm) at R\t = 2 which residual stresses are tensile at the outer-surface. For mid-section (50 mm), the compressive-stresses are located at the inner-surface and tensile-stresses are at the outer-surface. The peak compressive-stress is at the mid-layer towards inner-surface while the peak tensile-stress is at the mid-layer. For R/t = 2.5, the tensile-stresses reach the highest point near the edge (4 mm) at mid-layer (towards the inner-surface). For the compressive residual stress, it approaches its peak at mid-layer towards the inner-surface. For R/t = 3, similar stress distributions are found, the inner-surface has compressive-stresses and the outer-surface has tensile-stresses. The near edge (4 mm) reaches its peak tensile and compressive-stresses at the inner and outer-surfaces, respectively.

### 4.3. Duplex Stainless Steel

#### 4.3.1. Transverse

**Figure 17:**
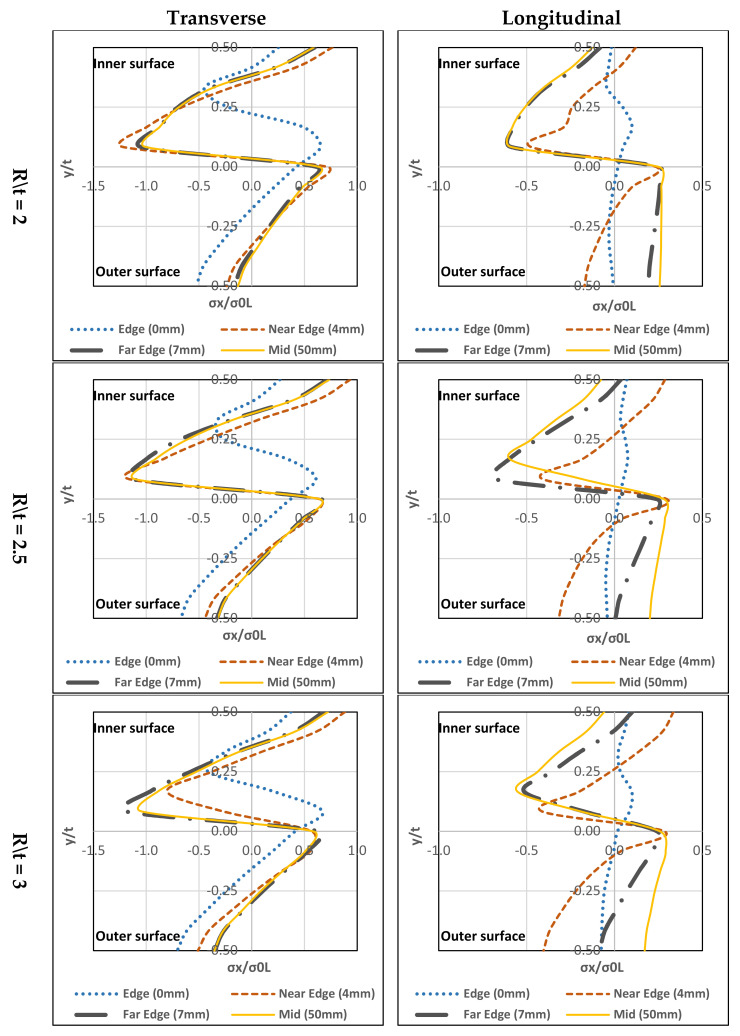
Through-thickness residual stresses distribution of duplex stainless steel.

In Figure 17, the three (R/t) ratios have similar stress distribution forms. The highest tensile residual stress is at the inner-surface and the maximum compressive residual stress is at the mid-layer (towards the inner). Except for the edge (0 mm), the stress distribution differs at the location with regards to the peak residual stresses (compressive residual stress is far from the mid-layer and closer to the inner-surface). Tensile residual stress is found at the upper mid-layer.

#### 4.3.2. Longitudinal

In Figure 17, there are a few differences between the three R/t ratios. For R/t = 2, at the near edge (4 mm), the inner-surface has tensile-stresses and the outer-surface has compressive-stresses. The others have compressive-stresses at the inner-surface and the tensile-stresses at the outer-surface. The peak compressive-stress is at the mid-layer towards inner-surface while the peak tensile-stress is at the outer-surface. For R/t = 2.5, the stress distribution form is tensile-stresses at the inner-surface and the compressive-stress is at the outer-surface except at the mid-section (50 mm), in which the compressive-stresses are at the inner-surface and tensile-stresses are at the outer-surface. The tensile-stresses reach the highest near edge (4 mm) at the inner-surface. However, the mid-section (50 mm) has closer tensile-stresses at the outer-surface. For compressive-stresses, the peak is at the mid-layer towards the inner-surface. For R/t = 3, similar stress distribution is seen, in which inner-surface has compressive-stresses, and outer-surface has tensile-stresses.

### 4.4. Lean Duplex Stainless Steel

#### 4.4.1. Transverse

**Figure 18:**
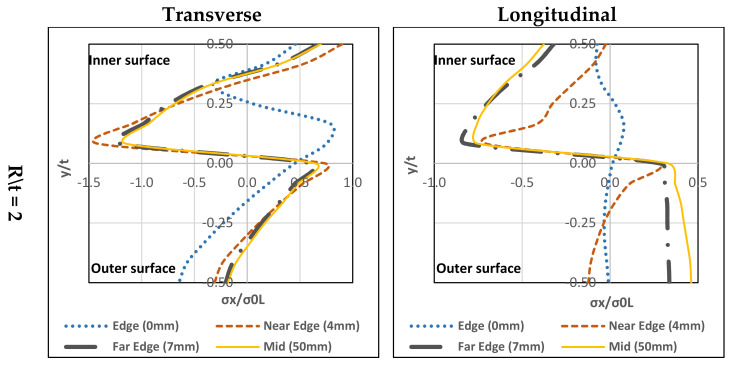

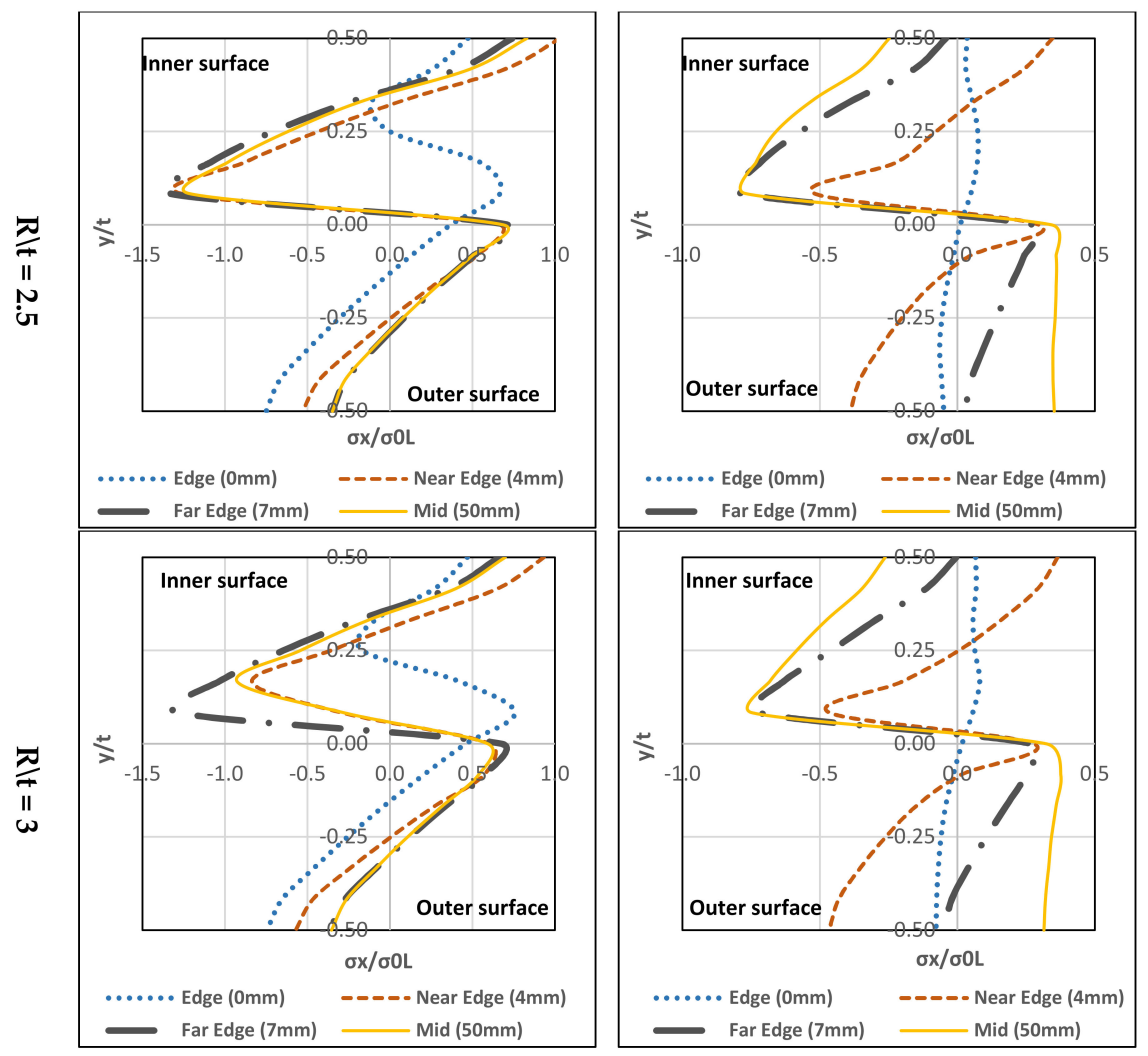
Through-thickness residual stresses distribution of lean duplex stainless steel.

In Figure 18, the three (R/t) ratios have similar stress distribution forms. The maximum tensile residual stress is found at the inner-surface and the maximum compressive-stress is at the mid-layer (towards the inner part). Except for the edge (0 mm), the difference in stress distribution with regards to its peak residual stresses (compressive residual stress is far from the mid-layer and closer to the inner-surface) can be seen. Tensile residual stress is found at the upper mid-layer). R/t = 2 has higher distribution values than that of R/t = 2.5 and 3. Near Edge (4 mm) at R/t = 2 has the highest compressive-stresses and it has the highest tensile-stresses at R/t = 2.5.

#### 4.4.2. Longitudinal

In Figure 18, there are a few differences between the three R/t ratios. For R/t = 2, at the near edge (4 mm) the inner-surface has tensile-stresses and the outer-surface has compressive-stresses. The others have compressive-stresses at the inner-surface and tensile-stresses at the outer-surface. The peak compressive-stress is at the mid-layer towards the inner-surface whereas the peak tensile-stress is at the outer-surface. For R/t = 2.5, tensile-stresses are at the inner-surface and compressive-stresses are at the outer-surface except for the mid-section (50 mm) the compressive-stresses are at the inner-surface and the tensile-stresses are at the outer-surface. The tensile-stresses reach the highest level near the edge (4 mm) at the inner-surface, however, for the mid-section (50 mm) the stress gets closer to the tensile-stresses at the outer-surface. For compressive-stresses, the peak is found at the mid-layer towards the inner-surface. For R/t = 3, similar stresses distribution is found, the inner-surface has compressive-stresses and the outer-surface has tensile-stresses. The mid-section (50 mm), has its peak tensile-stress at the mid-layer and the compressive-stresses exist at above the mid-layer (towards inner-surface).

### 4.5. Discussion

This parametric study intended to investigate residual stresses in four different stainless steel types (ferritic, austenitic, duplex and lean Duplex). The investigation predicted residual stresses along the corner zone of the four types with three different corner radius-to-thickness ratios. Material anisotropy for the four types was considered in the analysis.

The parametric study showed that all stainless steel types have the same stress through-thickness distribution form. On top of that, the R/t ratio’s effect is insignificant to the transverse residual stress distribution. Additionally, it can be seen that the transverse residual stress at the edge has a slight difference in stress distribution with regards to the peak residual stresses. On the other hand, a slight change to the R/t ratio can affect the longitudinal residual stress distribution. Figure 15, Figure 16, Figure 17 and Figure 18, show that the longitudinal residual stress distribution form at the edge differ significantly from the rest but in Figure 11c the longitudinal residual stresses have similar distribution form along the corner zone. This can be attributed that the effect of coiling-uncoiling is more pronounce than the R/t ratio.

Austenitic stainless steel has higher peak residual stresses (compressive and tensile) among the other stainless types. Ferritic, duplex and lean duplex showed a lot of similarities in stress distribution in terms of form and normalised stress values. In addition, the R/t ratio does not affect neutral axis shift for each type. Also, the austenitic stainless steel exceeds the yielding limit in the transverse direction for both compressive and tensile stresses while in the longitudinal direction for compressive stress only. The other three stainless steel types only exceed the yielding limit of their compressive stresses in the transverse direction while maintaining below the limit in the longitudinal direction for the three R/t ratio.

## 5. Conclusions

The 3D-FE technique presented in [24,25] was employed in this study to examine the residual stress behavior along the corner region and also in through-thickness direction of press-braked stainless steel sections giving due consideration to the influence of the material anisotropy. The outcomes was compared with two 2D-FE results from the literature: [14] which considered specimen’s material anisotropy and [17] that assumed material isotropy of the same specimen. This study also provided a parametric study for three corner radius-to-thickness ratios looking at the through-thickness residual stress distribution of four stainless steels (i.e., ferritic, austenitic, duplex, lean duplex). The following conclusions can be presented:

1. Residual stress is not linear along the corner region; peak longitudinal stress exists in the inner and outer surface due to the coiling-uncoiling process effect.
2. The coiling radius can be predicted using the 3D-FE (anisotropy) where maximum stresses exist in the inner and outer surfaces.
3. In simulation with material anisotropy, the neutral axis shift in the longitudinal residual stress distribution was lower than the one which neglected it. In addition, stresses near mid-surface are also higher.
4. Full finite element procedure incorporating coiling-uncoiling process shows higher longitudinal residual stress than the finite element simulation incorporating the coiling-uncoiling estimate using analytical method.
5. For transverse residual stress, the material effect is negligible.
6. The parametric study showed that all stainless steel types have the same stress through-thickness distribution form. Moreover, the effect of the R/t ratio is insignificant in all transverse residual stresses distribution. However, a slight change to the R/t ratio can affect the longitudinal residual stress distribution.
7. Austenitic stainless steel has higher peak residual stresses (compressive and tensile) among the other stainless steels.
8. Ferritic, Duplex and Lean Duplex showed many similarities in stress distribution in terms of form and normalised stress values.

## Figures and Tables

**Table 1:** Mechanical properties of UNS 31803 duplex stainless steel investigated in [34].

Specimen	E_0_ GPa	*σ*_0.01_ MPa	*σ*_0.2_ MPa	*e* (=*σ*_0.2_/E_0_)	*n*
*LC*	181.650	275	527	0.00290	4.6
*TC*	210.000	380	617	0.00294	6.2
*DC*	205.000	460	610	0.00298	10.6

**Table 2:** Material properties for FE parametric study (Longitudinal direction).

Type of Stainless Steel	$E_{0}\;\mathrm{GPa}$	$\sigma_{0.2}\;\mathrm{MPa}$	LT/LC	$\sigma_{u}\;\mathrm{MPa}$	*e* (=*σ*_0.2_/E_0_)	n
Ferritic [43]	197.0	280.0	LT	454.0	0.0014	-
Austenitic [44]	180.0	287.1	LT	596.5	0.0033	5.34
Duplex [5]	191.9	553.0	LC	-	0.0029	7.0
Lean Duplex [45]	191.0	486	LT	752	0.0025	12

**Table 3:** Engineering constants input for FE parametric study.

Type of Stainless Steel	E_1_	E_2_	E_3_	$\sigma_{0}$	$\tau_{0}$	$\sigma_{0,11}$	$\sigma_{0,22}$	$\sigma_{0,33}$	$\tau_{0,12}$	$\tau_{0,13}$	$\tau_{0,23}$
Ferritic [43]	199	199	197	289.7	167.3	295	295	280	167.3	170.3	167.3
Austenitic [44]	205.0	205.0	180.0	273.0	157.6	260.7	272.9	287.1	157.6	157.6	157.6
Duplex [5]	226.9	226.9	191.9	609.4	351.8	666.1	625.6	553	351.8	361.2	351.8
Lean Duplex [45]	208	208	191	520.1	300.1	540	540	486	300.3	311.8	300.3

## Notations

W_d_	Die opening
R	Intended-formed corner radius
t	Sheet thickness
D/t	The coil diameter/thickness ratio.
R/t	Corner radius-to-thickness ratios.
a, b	Material parameters.
σ_0D_	Through-thickness initial yield stresses in diagonal direction.
$\sigma_{0L}$	Initial yield stresses (taken as the 0.2% proof stresses) in the longitudinal (z) direction.
$\sigma_{0T}$	Initial yield stresses (taken as the 0.2% proof stresses) in the transverse (x) direction.
$\sigma_{0N}$	Initial yield stresses (taken as the 0.2% proof stresses) in through-thickness(y) direction.
σ_0.2_	0.2% proof of stress.
σ_1.0_	1.0% proof of stress.
σ_2.0_	2.0% proof of stress.
$\bar{\sigma }$	Equivalent true stress.
$\sigma_{t}$	True stress.
σ_0,ii_	Initial yield stress measured in the i-direction.
σ_0_	The reference yield stress.
σ_x_/σ_0L_	Normalized Stress.
$\sigma_{n}$	Nominal stress.
$\bar{\varepsilon }$p	Equivalent true plastic strain
$\varepsilon_{\mathrm{tp}}$	True plastic strain.
$\varepsilon_{n}$	Nominal strain respectively.
$\varepsilon_{t}$	True strain.
ε_x_	Residual transverse strain
y	The distance from center line to the surface
τ_0,ij_	Initial shear yield stress measured for the i-j plane.
$\tau_{0}$	The reference yield stress.
E_0_	The initial young modulus.
E_0.2_	Tangent modulus at the nominal yield stress.
E_1_, E_2_, E_3_	Elastic moduli in local coordinate system (1-transverse, 2-through thickness and 3-longitudinal directions).
ν_12_, ν_13_, ν _23_	Poisson’s ratios in local coordinate system.
G_12_, G_13_, G_23_	Shear moduli in local coordinate system.
LC	Longitudinal compression coupon
TC	Transverse compression coupon.
DC	Diagonal compression coupon.
n	Exponent for strain-hardening.
$n_{0.2,0.1}$	Strain hardening exponents the second stages.
R_ij_	State of plastic anisotropy is defined in ABAQUS in i-j plane.
y/t	Normalized thickness
